# Characterization of GABAergic Marker Expression in the Chronic Unpredictable Stress Model of Depression

**DOI:** 10.1177/2470547017720459

**Published:** 2017-08-03

**Authors:** Mounira Banasr, Ashley Lepack, Corey Fee, Vanja Duric, Jaime Maldonado-Aviles, Ralph DiLeone, Etienne Sibille, Ronald S. Duman, Gerard Sanacora

**Affiliations:** 1 Department of Psychiatry, Yale University School of Medicine, New Haven, CT, USA; 2 Campbell Family Mental Health Research Institute of CAMH, Toronto, Canada; 3 Department of Psychiatry, Pharmacology and Toxicology, University of Toronto, Toronto, Canada; 4 Department of Physiology and Pharmacology, Des Moines University, Des Moines, IA, USA

**Keywords:** depression, chronic stress, gamma-aminobutyric acid, GAD_67_, calcium-binding proteins, neuropeptides

## Abstract

**Background:**

Evidence continues to build suggesting that the GABAergic neurotransmitter system is altered in brains of patients with major depressive disorder. However, there is little information available related to the extent of these changes or the potential mechanisms associated with these alterations. As stress is a well-established precipitant to depressive episodes, we sought to explore the impact of chronic stress on GABAergic interneurons.

**Methods:**

Using western blot analyses and quantitative real-time polymerase chain reaction, we assessed the effects of five-weeks of chronic unpredictable stress exposure on the expression of GABA-synthesizing enzymes (GAD_65_ and GAD_67_), calcium-binding proteins (calbindin, parvalbumin, and calretinin), and neuropeptides co-expressed in GABAergic neurons (somatostatin, neuropeptide Y, vasoactive intestinal peptide, and cholecystokinin) in the prefrontal cortex and hippocampus of rats. We also investigated the effects of corticosterone and dexamethasone exposure on these markers *in vitro* in primary cortical and hippocampal cultures.

**Results:**

We found that chronic unpredictable stress induced significant reductions of GAD_67_ protein levels in both the prefrontal cortex and hippocampus of chronic unpredictable stress-exposed rats but did not detect changes in GAD_65_ protein expression. Similar protein expression changes were found *in vitro* in cortical neurons. In addition, our results provide clear evidence of reduced markers of interneuron population(s), namely somatostatin and neuropeptide Y, in the prefrontal cortex, suggesting these cell types may be selectively vulnerable to chronic stress.

**Conclusion:**

Together, this work highlights that chronic stress induces regional and cell type-selective effects on GABAergic interneurons in rats. These findings provide additional supporting evidence that stress-induced GABA neuron dysfunction and cell vulnerability play critical roles in the pathophysiology of stress-related illnesses, including major depressive disorder.

## Introduction

Major depressive disorder (MDD) is a common and debilitating mood disorder characterized by depressed mood and/or anhedonia. It is estimated that 17% of the population is affected by MDD, yet only approximately one third of patients achieve remission after treatment with current first-line therapeutic agents.^1,2^ Attempts to develop novel, improved antidepressant therapies have been hindered by a limited understanding of the precise neurobiological mechanisms underlying MDD. A deeper understanding of the neurobiology and pathophysiology associated with the disorder will facilitate the development of improved therapeutics by providing novel targets and potentially meaningful biomarkers.

Building upon early reports of reduced levels of gamma-aminobutyric acid (GABA) in the plasma and cerebrospinal fluid (CSF) of MDD patients,^3–5^ accumulating evidence indicates that GABA neurotransmission alterations are involved in the pathophysiology of MDD.^6,7^ Brain imaging and magnetic resonance spectrometry studies consistently demonstrate reduced GABAergic neurotransmission and GABA content in the prefrontal and occipital cortex of depressed patients,^8–10^ especially in patients with prominent anhedonia symptoms^10,11^ and treatment resistant depression.^12^

Postmortem studies have provided further evidence of GABAergic abnormalities related to MDD by identifying changes in GABAergic neuron-specific markers.^13,14^ GABA, the primary inhibitory neurotransmitter, is synthesized from glutamate in GABAergic interneurons by glutamic acid decarboxylases (GADs). GAD exists as two isoforms, GAD_65_ and GAD_67_, each performing distinct roles within the neuron. It is now recognized that GABAergic interneurons are highly heterogeneous in terms of multiple morphological, electrophysiological, and molecular properties.^15^ Co-expression of neurochemical markers, notably the calcium-binding protein parvalbumin (PV), the neuropeptide somatostatin (SST), and vasoactive intestinal peptide (VIP)/ionotropic serotonin receptor 3a, can be used to discriminate between three non-overlapping GABA interneuron populations.^16^ Dissecting the role of these cell subtypes has been useful for identifying molecular vulnerabilities across several neuropsychiatric disorders (reviewed in literature^17^). However, the involvement of these distinct populations in functional or pathological roles is only partially understood. Interneuron populations are further subdivided by co-expression of other calcium-binding proteins (e.g., calbindin (CB) and calretinin (CR)) and neuropeptides (e.g., neuropeptide Y (NPY) and cholecystokinin (CCK)), which together can help characterize vulnerable cell populations relevant to the pathological changes associated with MDD and other stress-related disorders.

Recent studies have identified a down regulation of mRNA levels of NPY, SST, GAD_67_, GAD_65_, and PV in the subgenual anterior cingulate cortex (sgACC) of depressed individuals but failed to demonstrate differences in CR expression.^18^ A second study found reduced SST mRNA and protein expression in the dorsolateral prefrontal cortex (PFC) of depressed patients but no changes in mRNA levels of GAD_65/67_, CR, or PV.^19^ At the protein level, expression of GAD_67_, but not GAD_65_, was reported to be decreased in the PFC.^14^ Contrary to findings from quantitative real-time polymerase chain reaction (qPCR) and immunoblotting studies, reductions in both GAD_65_– and GAD_67_– immunopositive neurons were found in this brain region.^20^ Furthermore, using stereological cell counting, the density and size of calbindin-immunoreactive (CB-IR) GABAergic interneurons in the PFC and occipital cortex were decreased.^13,21^ Similar reductions of calretinin-immunoreactive (CR-IR) GABAergic interneurons were reported in the PFC,^22^ while other studies have reported no change in the density of CB-IR, PV-IR, or CR-IR interneurons in the PFC or anterior cingulate cortex of MDD patients.^23,24^ The lack of consistency in findings from human postmortem studies is likely related to multiple variables, including treatment history and heterogeneity of the disorder, thus highlighting the need for preclinical studies that afford the opportunity to control for variables that may confound potentially informative cellular changes associated with the pathogenesis of the disorder.

Stress exposure is a major risk factor for the onset of depression.^25,26^ Previous studies have employed stress paradigms to probe pathological changes relevant to MDD within the GABAergic system in the rodent brain. Similar to clinical reports, preclinical stress studies have demonstrated reductions in GAD_65_ mRNA and protein levels following chronic mild stress exposure^27,28^ and chronic corticosterone (CORT) treatment,^29^ while others found no alterations in either GAD_65_ or GAD_67_ mRNA levels after chronic restraint stress.^29,30^ Chronic unpredictable stress (CUS) is a well-documented animal model that reproduces depressive-like behaviors in rodents that parallel human depressive symptoms (e.g., anhedonia and despair) and induces clinically relevant neurobiological changes.^31,32^ Previous studies using CUS have reported decreased density of CB-IR interneurons in both the PFC and hippocampus (HPC), while the density of PV interneurons was unchanged across brain regions.^33,34^ However, another study reported decreased PV cell number in the HPC following chronic psychosocial stress.^35^

Together, these clinical and preclinical studies indicate that GABAergic dysfunction is associated with MDD and chronic stress; although the findings have not been consistent relating to the specific components of the system that are altered. One limitation of these studies is that only a subset of GABAergic markers was examined. To address this, the current study pursued a more complete survey of the chronic stress effects on this system by investigating expression levels of several markers of GABAergic neurons in the rat PFC and HPC following CUS exposure. These regions are significant for their role in cognitive-emotional regulation, which is characteristically disrupted in MDD,^36,37^ as well as for the well-characterized stress- and MDD-related GABAergic deficits found in previous studies.^13,14,19,35,38^ We employed a CUS paradigm, previously validated in our laboratory, that replicates multiple behavioral and cellular features of MDD.^39,40^ Since chronically elevated CORT and glucocorticoid receptor activation are well-replicated biomarkers of chronic stress and implicated in vulnerability to MDD,^41,42^ we further investigated the cellular mechanisms involved in GABAergic changes and examined separately the effects of CORT or dexamethasone (DEX), a glucocorticoid receptor agonist, on primary cortical and hippocampal neurons *in vitro*. This approach tested a direct involvement of CORT on the expression levels of GABAergic markers.

## Materials and Methods

### Animals

Male Sprague-Dawley rats (Charles River Laboratories, 175–250 g) were housed and maintained on a 12-h light/dark cycle with food and water *ad libitum*, except when animals underwent food or light disruptions. All animal use and procedures were performed in accordance with NIH guidelines and Yale University Institutional Animal Care and Use Committee.

### CUS Paradigm

Animals were exposed to a random sequence of mild stressors (12 total, 2/day) for 36 days.^39,40^ The stressors included the following: 45°cage tilt, wet bedding, lights on overnight, lights off (3 h), food and water deprivation, isolation overnight, odor, 4℃ cold stress, swim stress (18℃), stroboscope overnight, crowded housing, and cage rotation. Home cage control (HCC) animals were handled daily but otherwise left undisturbed. On day 36, animals received 4℃ cold (1 h) and then cage rotation (1 h); 24 h after the final stressor, animals were sacrificed via decapitation (*n = *8/group for western blot and *n = *6/group for qPCR).

### Primary Culture

Cortices or hippocampi from rat E18 embryos were dissected and incubated in trypsin-EDTA (0.25%, Gibco, MA) for 10 min before being dissociated. Neurons were plated at 0.4 million cells per well in six-well poly-lysine-coated plates in DMEM (10% fetal bovine serum, Gibco). The following day, the medium was changed to serum-free medium DMEM containing neurobasal plus B27 (Gibco) and was changed every five days. Cells were cultured and maintained at 37℃, 5% CO2, and 95% humidity. On day 10, cells were exposed to various doses of CORT, DEX (Sigma, MO), or dimethylsulfoxide (DMSO) (vehicle) for 72 h.^43^ The doses of CORT (100 nM, 1 µM, 10 µM) and DEX (100 nM and 200 nM) used in this study were shown to induce oxidative stress at this time point.^44^ We confirmed the reduction in mitochondrial activity at these doses using MTT reduction assay (*n* = 4). All western blot experiments were performed in triplicate and repeated five to six times (*n = *5–6). The percentage of GABA neurons in primary culture was estimated using immunocytochemistry. On day 10, cells cultured on poly-lysine-coated slides were fixed with 4% paraformaldehyde (Sigma, MO) and incubated in 10% normal goat serum for 30 min after three phosphate-buffered saline (PBS)−0.1% Triton X-100 washes. Cells were then incubated with mouse primary antibody anti-GAD67 (Millipore, MA) overnight at 4℃, washed, and incubated with Alexa-565 goat secondary antibody anti-mouse (Millipore, MA) for 1 h. Slides were then washed, cover-slipped with DAPI vectashield containing (Vector Labs, CA), and visualized using fluorescence microscopy.

### Western Blot Analysis

Prefrontal cortices and hippocampi of rats were dissected and immediately frozen. Whole homogenates were sonicated in protein lysis buffer containing 50 mM Tris-HCl, 150 mM NaCl, 1 mM NaVo3, 10 mM NaF, 1X protease inhibitor cocktail, 1% Triton X-100, and 0.1% SDS. Protein concentrations were measured using Pierce BCA Assay Kit (Thermo Scientific, IL). Proteins were separated by sodium dodecyl sulphate-polyacrylamide gel electrophoresis and then transferred to 0.2 µm nitrocellulose membranes. After three washes with PBS + 0.1% Tween-20 (PBS-T) and blockade in 5% milk/PBS-T for 1 h, membranes were incubated with primary antibodies overnight, except for anti-glyceraldehyde 3-phosphate dehydrogenase (GAPDH; Advanced Immunochemical, CA) which was incubated for 1 h; 20 µg of protein was separated using 7.5% TGX Gels (Biorad, CA) for mouse anti-GAD67 and anti-GAD65, rabbit anti-CB, and goat anti-CR (Millipore, MA). For rabbit anti-PV (Novus Biologicals, CO), 40 µg of protein was separated using 4% to 20% gels. Primary antibodies were used at a 1:1000 dilution in 2% BSA/PBS-T. After three washes, membranes were incubated in peroxidase-labeled secondary antibody (Vector; 1:10,000) in 5% milk/PBS-T for 1 h room temperature. Protein bands were visualized using enhanced chemiluminescence. Subsequently, membranes were incubated for 30 min in stripping buffer (Thermo Scientific) and incubated in mouse anti-GAPDH (1:20,000; loading control).

For the in vitro studies, 72 h after treatment with either CORT or DEX, cultured cortical and hippocampal neurons were scraped, collected in lysis buffer (same as above), and sonicated; 10 µg of protein from cultured cells was used for western blotting, as described above.

### Quantitative Real-Time PCR

Total RNA (500 ng for rat tissue, 225 ng for primary neurons) was extracted with RNAqueous kit (Ambion, TX) and reverse-transcribed into cDNA in 20 µL reactions using oligo-dT primers (Genisphere, PA). qPCR was performed using a hot-start SYBR Green (Qiagen, CA) method with ABI7900 instrument (Applied Biosystems, CA), followed by melt-curve analysis to further verify specificity. Forward and reverse primers for genes of interest were designed using Primer3 v.0.4.0 software (http://frodo.wi.mit.edu/cgi-bin/primer3/primer3_www.cgi; Whitehead Institute for Biomedical Research, MA). Primer specificity was additionally verified using BLAST Interface software (NCBI). Gene expression fold change was calculated using ΔΔCt (Ct = cycle number at threshold) analytical method that includes normalization against the house-keeping gene *GAPDH*. Sequences of qPCR primers (forward and reverse) used are as follows:

*GAPDH*: 5′-cgtggaagggctcatgaccacag-3′ and 5′-caccagtggatgcagggatgatg-3′

*SST*: 5′-gcccaaccagacagagaacgatgc-3′ and 5′-gctgggttcgagttggcagacctc-3′

*NPY*: 5′-ctgctcgtgtgtttgggcattctg-3′ and 5′-cagtgtctcagggctggatctcttgc-3′

*VIP*: 5′-gctgcagttcgaaggagcaggtg-3′ and 5′-gcctggcatttctggacacatc-3′

*CCK*: 5′-cgcagccggtagtccctgtagaag-3′ and 5′-ctgctggatgtatcgggctagcagtg-3′

*PV*: 5′-ggcgataggagcctttactg-3′ and 5′-tcttcacatcatccgcactc-3′

### Statistical Analysis

All statistical analyses were performed using StatView (SAS Institute, ON). Protein bands were analyzed by densitometry with ImageJ (NIH), and protein levels were normalized to GAPDH. Real-time-(PCR) data were analyzed according to the ΔΔCT method, as previously shown.^45^ To determine statistical significance between CUS and HCC animals, a *t*-test was performed. Because this study was intended to be an exploratory survey of a large range of biomarkers, there was no correction for multiple comparisons. *In vitro* western blots were analyzed using a one-way analysis of variance followed by a Fisher’s protected least significant difference *post-hoc* analysis. All data are expressed as average fold change compared to the HCC groups (*in vivo*) or DMSO-treated cultures (*in vitro*).

## Results

### Effects of CUS on GAD_65_ and GAD_67_

**Figure 1.**
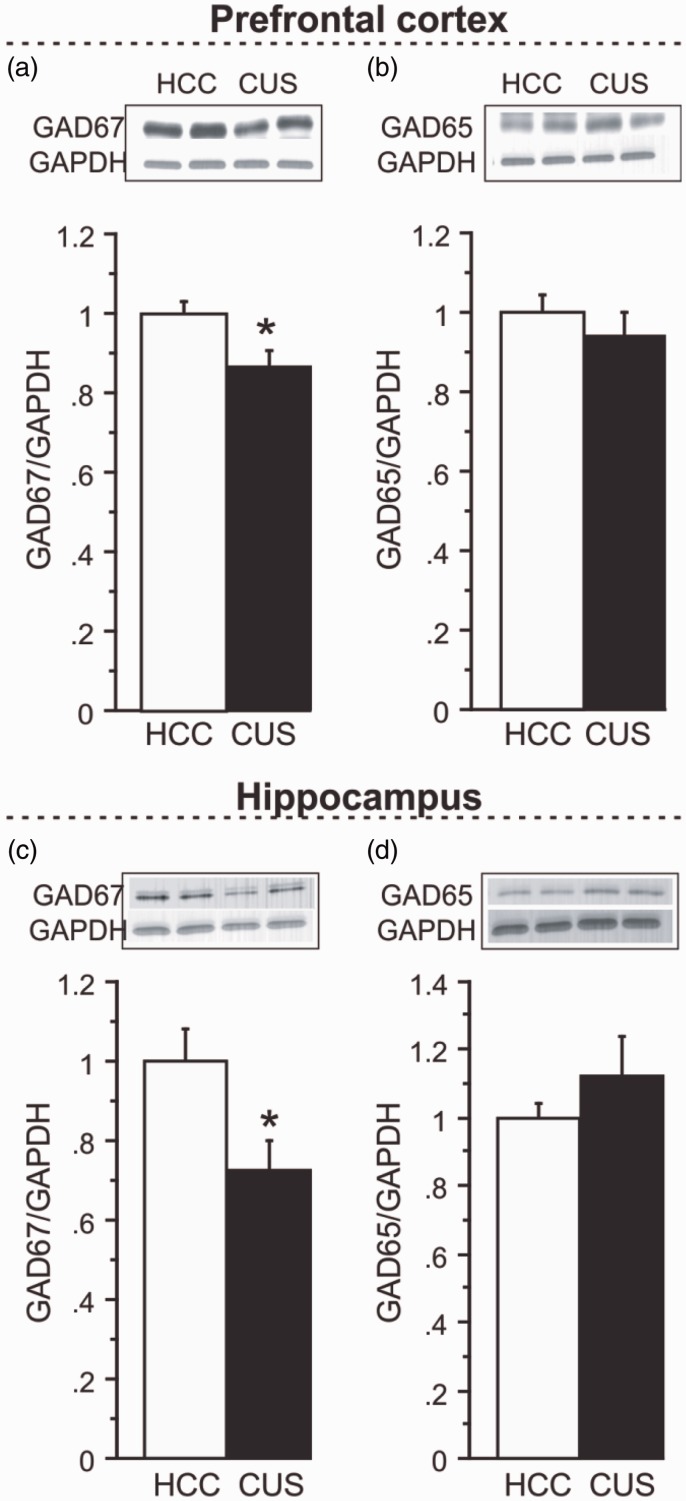
Effects of chronic unpredictable stress (CUS) on GAD_65_ and GAD_67_ protein expression. (a) GAD_67_ and (b) GAD_65_ protein expression following CUS compared to home cage controls (HCC) in the PFC. (c) GAD_67_ and (d) GAD_65_ protein expression in the HPC. Levels of proteins were normalized to GAPDH. For each marker, a representative immunoblot and its respective GAPDH blot are illustrated. Results are expressed as fold change compared to HCC and displayed as means ± *SEM*. *p < 0.05.

Following 36 days of CUS, a significant 13% decrease in GAD_67_ protein levels was identified by western blot analysis in the PFC of CUS-exposed animals, compared to HCCs (Figure 1(a); *n = *8/group, *p < *0.05). PFC GAD_65_ levels were not significantly altered by CUS exposure (Figure 1(b)). In the HPC, CUS exposure induced a 27% decrease in GAD_67_ protein expression (Figure 1(c); *p < *0.05). Similar to findings in the PFC, GAD_65_ levels were not reduced in the HPC following CUS exposure (Figure 1(d)).

### Effects of CUS on Calcium-Binding Proteins Specific to GABAergic Interneurons

**Figure 2.**
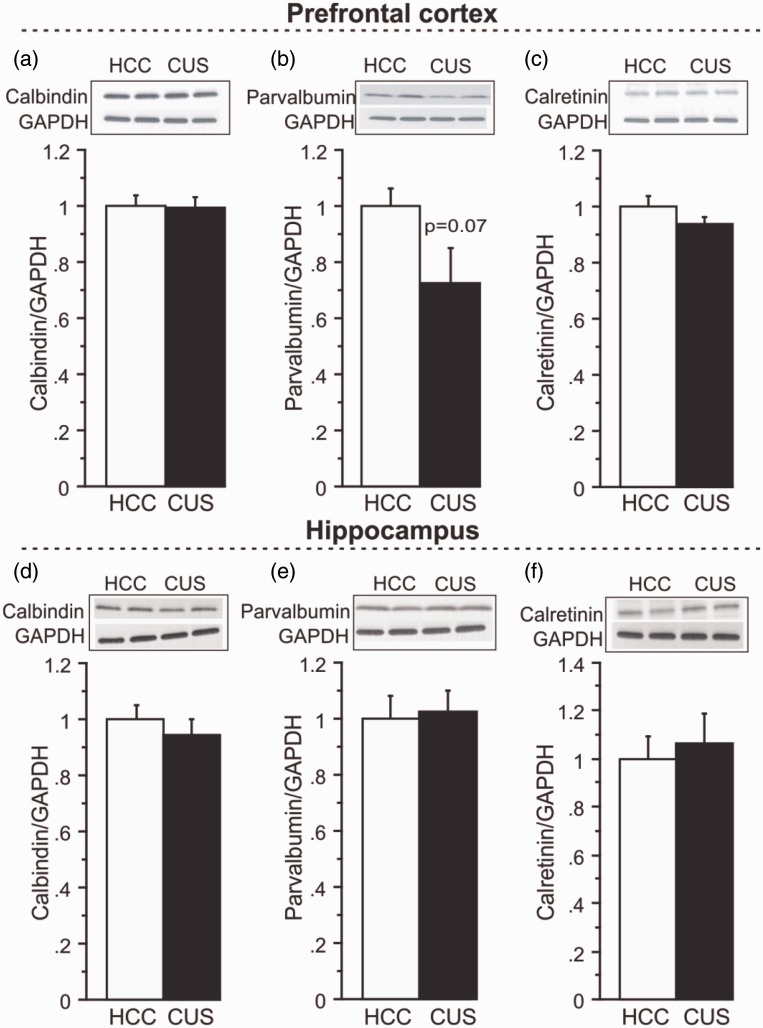
Effects of CUS on calcium-binding proteins. For each marker, a representative immunoblot and its respective GAPDH blot are illustrated. (a) CB, (b) PV, and (c) CR protein expression following CUS compared to HCC in the PFC. (d) Levels of CB, (e) PV, (f) and CR protein expression in the HPC following CUS compared to HCC. Levels of proteins were normalized to GAPDH. Results are expressed as fold change compared to HCC and displayed as means ± *SEM*. *p < 0.05.

To examine whether specific GABAergic interneuron subtypes are preferentially affected by CUS, protein levels of calcium-binding proteins co-expressed in GABAergic neurons were analyzed by western blot analysis. In the PFC, no significant changes in CB (Figure 2(a)), PV (Figure 2(b)), or CR (Figure 2(c)) protein expression were detected following CUS, although there was a robust 27% decrease in mean PV protein expression in the PFC of CUS compared to HCC animals that did not reach the predetermined statistical criteria of significance (Figure 2(b); *n = *8/group, *p = *0.07), despite a relatively large effect size (Table 1). There were no significant changes detected in CB (Figure 2(d)), PV (Figure 2(e)), or CR (Figure 2(f)) protein levels in the HPC following CUS.

**Table 1.** Summary table illustrating direction of changes, 95% confidence intervals (CI) and effect size of the effects of CUS on the GABAergic marker expression levels in the prefrontal cortex and the hippocampus.

Prefrontal cortex					Hippocampus			
Marker	Direction of change (*p < 0.05)	HCC [95% CI]	CUS [95% CI]	Effect size (*d*)	Direction of change (*p < 0.05)	HCC [95% CI]	CUS [95% CI]	Effect size (*d*)
Western blot								
GAD_67_	↓*	[0.98–1.02]	[0.84–0.89]	1.23	↓*	[0.95–1.05]	[0.91–0.98]	1.31
GAD_65_	NS	[0.97–1.03]	[0.90–0.98]	0.41	NS	[0.97–1.03]	[0.67–0.78]	0.48
PV	NS	[0.94–1.04]	[0.64–0.81]	0.93	NS	[0.94–1.06]	[1.04–1.20]	0.08
CB	NS	[0.97–1.03]	[0.97–1.02]	0.09	NS	[0.96–1.04]	[0.97–1.08]	0.32
CR	NS	[0.97–1.03]	[0.92–0.96]	0.65	NS	[0.94–1.06]	[.98–1.15]	0.19
RT-PCR								
CCK	NS	[0.96–1.12]	[1.04–1.11]	0.13	NS	[0.95–1.17]	[0.73–0.84]	0.88
VIP	NS	[0.96–1.07]	[0.90–0.93]	0.68	NS	[0.96–1.12]	[0.73–0.88]	0.8
SST	↓*	[0.96–1.11]	[0.68–0.82]	1.13	NS	[0.94–1.20]	[0.73–0.86]	0.84
NPY	↓*	[0.96–1.10]	[0.73–0.82]	1.17	NS	[0.95–1.19]	[0.80–0.86]	0.74

HCC: home cage control; CUS: chronic unpredictable stress; PV: parvalbumin; CB: calbindin; CR: calretinin; RT-PCR: real-time-polymerase chain reaction; CCK: cholecystokinin; VIP: vasoactive intestinal peptide; SST: somatostatin; NPY: neuropeptide Y; NS: non significant.

### Effects of CUS on Neuropeptides Specific to GABAergic Interneurons

**Figure 3.**
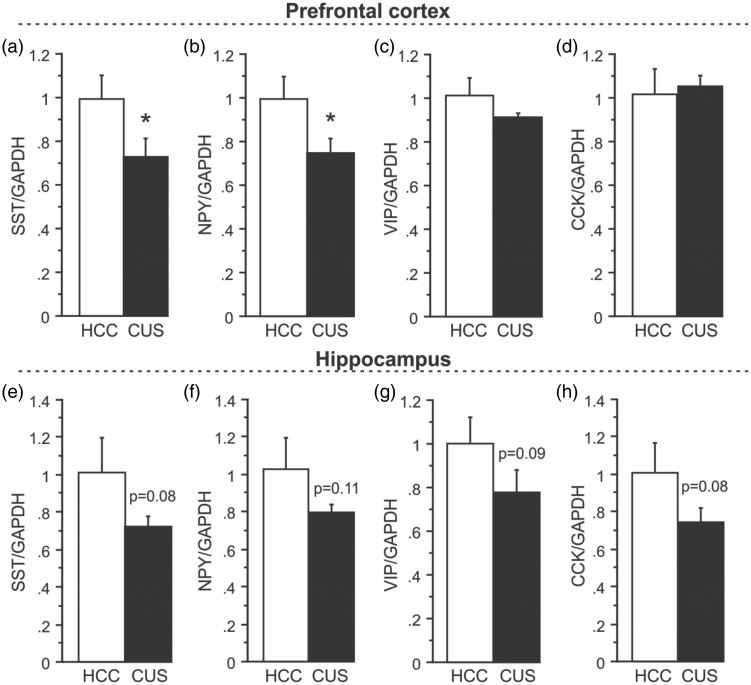
Effects of CUS on neuropeptides specific to GABAergic interneurons. (a) mRNA levels of SST, (b) NPY, (c) VIP, and (d) CCK following CUS compared to HCC in the PFC. (e) mRNA levels of SST, (f) NPY, (g) VIP, and (h) CCK after CUS exposure compared to HCC in the HPC. Gene expression was normalized to GAPDH. Results are expressed as fold change compared to HCC and displayed as means ± *SEM*. *p < 0.05.

To further investigate interneuron subtype-specific vulnerability to CUS exposure, gene expression levels of neuropeptides co-expressed in GABAergic interneurons were analyzed. qPCR was employed due to a lack of specificity of available antibodies and/or low levels of neuropeptide protein in the brain. Gene expression analysis revealed a 28% decrease in SST mRNA levels in the PFC of CUS-exposed compared to HCC animals (Figure 3(a); *n = *6/group, *p < *0.05). CUS also induced a 26% decrease in cortical mRNA expression of NPY (Figure 3(b); *p < *0.05). However, no clear alterations in either VIP (Figure 3(c)) or CCK (Figure 3(d)) mRNA levels were detected. In the HPC, gene expression analysis demonstrated an overall trend towards decreased mRNA levels of all neuropeptides examined: 30% for SST (Figure 3(e); *n = *6/group, *p = *0.08), 24% for NPY (Figure 3(f); *p = *0.11), 23% for VIP (Figure 3(g); *p = *0.09), and 28% for CCK (Figure 3(h); *p = *0.08), but none reached statistical significance (predetermined alpha of *p* < 0.05) despite relatively large effect sizes (Table 1).

### Effects of CORT or DEX Exposure on GABAergic Neurons In Vitro

**Figure 4.**
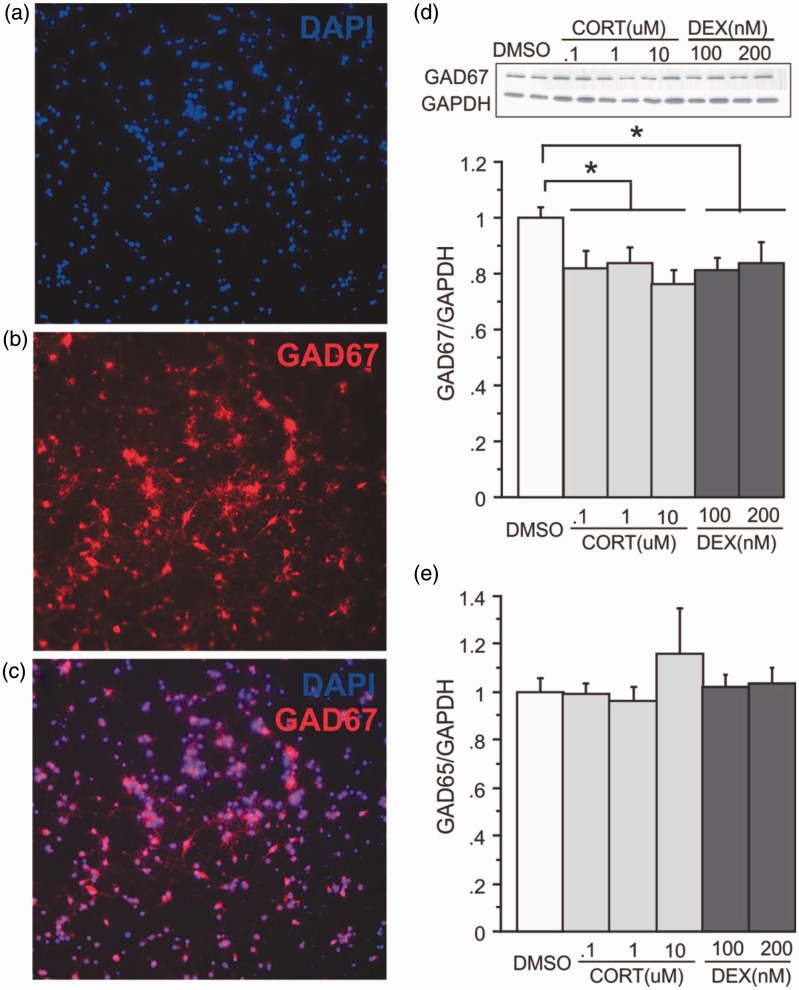
Effects of corticosterone or dexamethasone on GABAergic neurons *in vitro*. Illustration of (a) DAPI-positive cells from cortical primary neuronal culture, where (b) cells were labeled with GAD_67_ using immunocytochemistry and visualized with fluorescence microscopy to show co-localization of both markers (merge, c). (d) GAD_67_ and (e) GAD_65_ protein expression following 72 h exposure to various doses of corticosterone (CORT) or dexamethasone (DEX), compared to DMSO. For GAD_67_, a representative immunoblot and its respective GAPDH blot are illustrated. Levels of proteins were normalized to GAPDH. Results are expressed as fold change compared to vehicle treatment and displayed as means ± *SEM*. *p < 0.05.

We also examined the consequence of direct application of CORT or DEX, a synthetic, high affinity glucocorticoid receptor agonist, on cortical DIV10 neuronal cultures. On day 10, primary cortical cultures (containing approximately 30%–40% GABAergic neurons, Figure 4(a)) were exposed to CORT (0.1 µM, 1 µM, or 10 µM) or DEX (100 nM or 200 nM) for 72 h. The CORT doses and time-point were based on previous reports and induced a reduction in mitochondrial activity^44^ as confirmed using MTT reduction assay (DMSO: 100 ± 0.2; CORT0.1: 98.5 ± 6.3; CORT1: 83.9 ± 3.2*; CORT10: 79.6 ± 5.4*, *p < 0.05 vs. DMSO). Similar reductions were found following DEX exposure (DEX100: 82.2 ± 2.5*; DEX200: 80.4 ± 1.3*; *p* < 0.05 compared to DMSO). All doses of CORT and DEX (72 h) induced approximately a 30% reduction of GAD_67_ protein expression compared to DMSO (Figure 4(a); *n = *6/group, *F*(5, 25) = 8.076, *p < *0.0001) but did not alter expression of GAD_65_ (Figure 4(b)). There was no significant change in PV, CB, or CR protein expression or neuropeptide mRNA expression with either CORT or DEX exposure (data not shown). However, we again found a non-significant 21% and 24% decrease in PV mRNA expression with DEX 100 nM and 200 nM, respectively. Primary hippocampal neuronal cultures from rat did not show altered protein expression of GAD_65_ or GAD_67_ when exposed to CORT or DEX (*n = *3, data not shown), suggesting that intact hippocampal microcircuitry *in vitro* is necessary to investigate the direct (or local) actions of CORT and DEX on GABA neuron makers. Therefore, CB, PV, CR, SST, NPY, VIP, and CCK changes were not studied in hippocampal primary neuron culture.

## Discussion

Our results demonstrate evidence of CUS-induced alterations in GABAergic markers in rats. Specifically, GAD_67_, but not GAD_65_ expression levels are decreased in both the PFC and HPC following 36 days of CUS exposure. Similarly, only GAD_67_ protein levels were decreased after CORT and DEX exposure in primary cortical neuron culture. We failed to detect any significant changes in calcium-binding protein expression following CUS in this relatively small sample; however, there was a trend for a reduction of PV protein levels in the PFC. Significant decreases in SST and NPY mRNA were found in the PFC, but no statistically significant change in either CCK or VIP were observed in this region. In the HPC, while no change met the criteria for predetermined statistical significance, a trend towards reduced expression of all neuropeptide markers was found. In sum, our exploratory survey suggests a selective decrease in GABAergic markers following CUS, with more prominent deficits in the PFC compared to the HPC.

Numerous reports of reduced GABA levels in the brain of MDD patients have been published over the last 30 years.^7^ More recently, studies using technologies such as transcranial magnetic stimulation^46–49^ and neuroimaging^50^ have provided strong evidence of functional consequences related to GABAergic abnormalities in the MDD brain. These reductions are potentially the consequence of decreased levels of GAD_65/67_, the enzymes responsible for GABA synthesis from glutamate, as reported in several studies and across brain regions.^14,20,51,52^ In this study, we demonstrate a decrease in GAD_67_ protein levels in both the PFC and HPC following CUS. This is consistent with postmortem studies showing reductions in GAD_67_ protein levels in the PFC of MDD subjects.^14^ Using the same brain bank of MDD subjects, other studies report additional genes associated with depression pathology that are similarly affected in our CUS model. For example, hippocampal increases in expression of mitogen-activated protein kinase phosphatase-1, a negative regulator of mitogen-activated protein kinase intracellular signaling,^53^ or altered expression of synaptic proteins or GATA1, a transcription factor involved in synaptogenesis.^54^ Combined with evidence of decreased GAD_67_ expression in the PFC of CUS-exposed animals in this study, comparable to pathology in humans,^14^ these changes further support CUS as a valid rodent model to explore the pathogenic and pathophysiological processes associated with depression. Despite these similarities, current results are not consistent with all postmortem studies. Indeed, others report unchanged GAD_67_ mRNA levels in the dlPFC of MDD patients.^19^ Conversely, another study reported reductions in both GAD_65_ and GAD_67_ mRNA levels in the sgACC of depressed patients.^13^ These discrepancies may be related to level of analysis (protein vs. mRNA), the brain regions analyzed (dlPFC vs. sgACC), underpowered sample sizes, or sex differences (e.g., in the latter study, GAD_67_ mRNA reductions were found in only male subjects, which could mask potential decreases in mixed cohorts).

GAD_65_ and GAD_67_ are functionally distinct isoforms in the brain. GAD_67_ is localized to cell bodies and proximal dendrites and is thought to synthesize metabolic or cytosolic GABA, whereas GAD_65_ is found in axon terminals and believed to mediate fast conversion of glutamate to GABA.^55^ Consistent with this functional distinction, mice lacking the GAD_65_ isoform show few behavioral impairments and no change in GABA levels,^56^ whereas mice lacking GAD_67_ show overall decreased GABA content.^57^ The decrease of GAD_67_ expression found in humans^14^ may explain the decrease in GABA content consistently reported in 1H-MRS studies.^8–12^ Furthermore, decreased GABA concentration was found in the PFC and HPC of rodents exposed to stress,^58–60^ supporting the hypothesis that chronic stress causes lower GAD_67_ expression, in turn affecting total GABA content.

The mechanisms underlying GABA content reductions are not fully understood. Following CORT exposure, we found a decrease in mitochondrial metabolism (MTT assay) and a reduction in GAD_67_ protein expression in cortical primary cultures. Similar changes were induced by DEX exposure, suggesting that these effects were directly mediated by glucocorticoid receptor activation. Interestingly, GAD_67_ expression was unchanged in hippocampal primary cultures following CORT or DEX exposure. This difference may be due to a disruption of cortical microcircuitry *in vitro,* suggesting an indirect effect of stress or CORT on HPC GAD_67_ expression *in vivo.* Future studies using *in vivo* infusion of CORT or DEX within the HPC or using organotypic hippocampal culture may answer the question of whether an indirect, but local, action of CORT and/or glucocorticoid receptor activation affects GABAergic marker expression levels in this brain region.

As modeled in the current study, environmental or biological mediators of the stress response have the potential to confer changes at the cellular and subcellular level through mechanisms that have not been fully elucidated. Mitochondria oxidative stress and endoplasmic reticulum-related stress have recently been implicated in the pathophysiology of MDD^61–64^ and recapitulated in rodent models as biomarkers of chronic stress-related damage.^65,66^ Disruption of the function of these organelles is linked to altered energy balance and protein translation, potential candidate mechanisms underlying oxidative stress and cellular atrophy in MDD.^67,68^ Reports have also shown selective vulnerability of SST-positive GABA interneurons and involved chronic stress-induced altered proteostasis and endoplasmic reticulum stress as potential cellular mechanisms for GABA cell vulnerability.^65^ Further, social isolation in a mouse model of schizophrenia identified a selective vulnerability to oxidative stress in PV interneurons.^69^ In support of a cell type-specific vulnerability hypothesis, strong co-localization of neuronal nitric oxide synthase, an enzyme involved in the production of reactive oxygen species, has been identified in cortical SST, NPY, and CR populations.^70^ Although in this study, the *in vitro* cortical neuropeptide expression levels were not altered by CORT or DEX administration, it is likely that the time-frame (72 h exposure) greatly underestimates the chronic and progressive trajectory of changes present in both *in vivo* rodent stress models and human MDD.

Numerous postmortem studies,^13,18,21^ as well as rodent stress reports,^33,34^ describe changes in expression patterns of calcium-binding proteins. In the present study, we found no statistically significant changes in expression of PV, CB, or CR protein levels in the PFC, although there was evidence of moderate to large effect sizes for PV and CR. The absence of change in CR protein expression is consistent with the majority of studies in the human MDD literature.^18,19,24^ However, a review of several studies using the Stanley Brain Bank suggests a decrease in CR-IR interneurons in the dlPFC of MDD patients.^22^ Studies investigating changes in CB interneurons have been conflicting. Although our study is consistent with some postmortem findings reporting no change in the PFC,^23,24^ other postmortem^13,21^ and rodent stress studies show reductions in CB-IR interneurons.^33,34^ These changes may be limited to sub-regions of the cortex, since reductions in CB-IR interneurons were only significant in the Brodmann area (BA) 9 of the PFC and not in BA47.^13^ However, the same study showed a strong trend towards decreased PV-IR neuron density in BA47 and not in BA9, potentially reflecting the trend towards reduced PV protein levels observed here in the whole PFC. These trends toward changes in PV-IR neuron density or protein levels may be indicative of a potential susceptibility to stress within this cell type population. This is consistent with recent findings indicating that PV neurons are more vulnerable to chronic stress compared to other types of GABAergic neurons,^35,71,72^ highly susceptible to oxidative stress,^69^ and a preferential target across rodent models of schizophrenia, where reduced PV neurons are consistently reported.^73^

Neuropeptides co-expressed in GABAergic interneurons have been implicated in mood disorder pathology. Several clinical and postmortem studies^19,74,75^ in addition to depression-related animal model studies^76–78^ have demonstrated neuropeptide alterations. In the present study, we found a trend towards reductions in SST, NPY, VIP, and CCK mRNA levels in the HPC. The lack of significance (yet large effect size) could be due to the cellular heterogeneity associated with the various hippocampal subfields or the functional heterogeneity of this brain region (dorsal vs. ventral HPC). In the PFC, we demonstrated that CUS induced a significant decrease in SST and NPY mRNA levels in the PFC. This is consistent with reports of reduced CSF GABA levels, and that CSF SST levels are decreased in depressed patients,^79,80^ although more recent studies have not always replicated these findings.^81,82^ SST mRNA and protein levels are also reduced in the PFC and sgACC of MDD patients.^18,74^ Interestingly, SST neuron silencing has been shown to induce depressive-like behaviors,^83^ whereas infusion of SST into the cortex, septum, amygdala, or i.c.v.^77,84,85^, and infusion of SST agonists into the HPC, is sufficient to produce antidepressant-like effects in rodent models.^86^ Together, these findings suggest that SST may be dually involved in depressive pathology and treatment.

Studies investigating NPY levels in MDD have been inconsistent, whereby some report reduced NPY levels in the CSF^75,82^ and decreased NPY content in postmortem tissue,^87^ and others fail to replicate these findings.^88,89^ More consistently, preclinical studies using various stress paradigms showed reductions in NPY transcripts across multiple brain regions, including the HPC, PFC, and amygdala,^78,90,91^ whereas NPY infusion into the CA3 region of the HPC, or i.c.v., produces antidepressant-like effects.^92,93^ Taken together, these data highlight the utility of animal models and validate our findings of reduced SST and NPY transcripts in the PFC following CUS, along with the involvement of these neuropeptides or GABAergic cell types co-expressing these markers in depression or antidepressant action.

Finally, we found no statistically significant change in CCK or VIP mRNA levels in the PFC or HPC. This was in contrast with the reported increased in CCK levels in the cortex^94,95^ and decreased VIP expression in specific sub-regions of the HPC^96^ following chronic stress.

Although overall the results of this exploratory survey are consistent with the most recent literature implicating GABA neurons dysfunction in the response to stress, there are several limitations to consider including the approach employed, small-sample sizes that impeded correction for multiple comparisons and the lack of correlates with behavioral outcomes. Indeed, we opted for the use of the CUS paradigm and analysis of the protein/RNA expression changes as a proxy of cell functionality. Further studies will be required to demonstrated that the observed changes can be replicated and generalized in/to other rodent stress models and translate into reduced function using electrophysiology and/or correlate with behavioral deficits.

In summary, our results suggest that the PFC and HPC are susceptible to chronic stress-induced alterations of GABAergic interneurons, at multiple levels affecting GABA-synthesizing enzymes and co-expressed neurochemical markers. Further, these results suggest evidence of a cellular vulnerability to chronic stress, particularly in GAD_67_/SST/NPY-expressing cortical interneurons, supporting recent findings implicating dysfunction of this interneuron subtype in the emergence of depressive-like behaviors,^65,83^ and remediation of function as potentially underlying antidepressant-like effects.^97,98^ Characterizing the underlying mechanisms responsible for this vulnerability will advance our understanding of MDD and stress-related illnesses. Future studies employing tools designed to specifically alter these cell types (e.g., transgenic mouse lines, chemogenetics, or optogenetics) can help identify whether these cell-specific dysfunctions are a cause or consequence of depression, and their contribution to the symptoms of MDD,^99,100^ and may lead to novel therapeutic targets for the treatment of depression.
